# Application of Ultrasound Diagnosis Technology Based on Statistical Analysis in Rehabilitation Treatment of Shoulder Sports Injuries

**DOI:** 10.1155/2021/4867850

**Published:** 2021-12-22

**Authors:** Huiyu Duan, Shenglong Xun, Yusong Teng, Gong Zhang

**Affiliations:** ^1^Department of Physical Education, Inner Mongolia University of Technology, Hohhot 010051, China; ^2^Department of Physical Education, Inner Mongolia University of Technology, Hohhot 010051, China; ^3^School of Physical Education, Liaoning Normal University, Dalian 116029, China; ^4^Institute of Foreign Language Inner Mongolia University of Technology, Hohhot 010051, China

## Abstract

**Objective:**

This study uses statistics to analyze the diagnostic significance of ultrasonic exploration for rotator cuff injuries.

**Methods:**

For this study, 50 patients with rotator cuff injury or shoulder impingement syndrome admitted to the hospital from September 2017 to January 2019 were selected as the targets of this discussion. The general clinical materials of the patients were retrospectively analyzed, and ultrasound was performed for them. The results of the examination and arthroscopy are compared with the final pathological results.

**Results:**

The diagnostic sensitivity of ultrasound in the diagnosis of partial rupture and complete rupture of the supraspinatus tendon was 100%, and the specificity was 55.55%. The diagnostic sensitivity of partial rupture and complete rupture of the subscapular tendon was 100%, and the specificity was 42.8%; there was no significant difference compared with the joint mirror examination, and there was no statistical significance.

**Conclusion:**

The thesis adopts ultrasound exploration for patients with rotator cuff injuries with high diagnostic sensitivity. It is a reliable and effective clinical diagnosis method, which is worthy of clinical application and promotion.

## 1. Introduction

In clinical practice, shoulder pain and limited shoulder mobility are widespread, second only to back pain and knee pain, and the third-highest incidence of motor system pain diseases, accounting for about 16%∼26%. Rotator cuff injury is the leading cause of shoulder pain, which occurs in about 65% to 70% of patients. With the increase of age, the incidence of rotator cuff injury increases year by year, especially for people over 70 years old, more than 50% have partial or full-thickness rotator cuff injury, but some have no apparent symptoms. Therefore, early diagnosis and treatment of rotator cuff injuries are essential. Otherwise, the injury area will gradually increase, and the pain will gradually increase as well. A damaged rotator cuff is often accompanied by irreversible steatosis. Once the tendon degeneration occurs, the possibility of recurrence after rotator cuff repair increases to 94%. Ultrasonography can be used as the tool of choice for the early diagnosis of shoulder joint diseases. Ultrasound examination can perform multidirectional imaging of the shoulder joint and its surrounding soft tissues and can dynamically observe the movement of various muscles and ligaments in real-time without the risk of radiation. This makes ultrasonography's role in the diagnosis and treatment of shoulder joint diseases more and more critical. The most common rotator cuff and other tissue diseases found in ultrasound examinations include rotator cuff and biceps, prolonged head tendon abnormality, glenohumeral joint, acromioclavicular joint injury, and other soft tissues surrounding the shoulder joint. In this study, to analyze and study the diagnostic significance of ultrasound exploration for shoulder joint injury, patients with rotator cuff injury or shoulder impingement syndrome admitted to the hospital within two years were selected for clinical discussion [1]. The report is summarized and detailed as follows.

## 2. Materials and Methods

### 2.1. General Information

For this study, 50 patients with shoulder impingement syndrome or rotator cuff injuries who were admitted to hospital from September 2017 to January 2019 were selected as this study's subjects. There were no injury cases that did not reach the injury level, and the patients' general clinical information was analyzed retrospectively. Among them, there were 27 male patients and 23 female patients. The oldest was 74 years old, and the youngest was 23 years old. The patients' average age was 43.5 ± 11.5 years; the longest course was 16 years, and the shortest three days, with an average of 5.54 ± 1.21*a*. All patients underwent ultrasound exploration and joint mirror inspection. The interval between ultrasound exploration and arthroscopic shoulder surgery was 0 to 106 days, and there was no history of shoulder surgery.

### 2.2. Method

Ultrasonic inspection instrument selection: produced by GE, model LogiqE9, probe frequency is 6–12 MHz.

Exploration measures are as follows: When all patients undergo ultrasonic exploration, they sit on the examination chair, and the operator performing ultrasonic exploration stands in front of the patient. When starting the examination, the biceps button's position is fixed and used as the index point. The inner side is moved to obtain the subscapular muscle sonogram. When the upper arm is very externally rotated, the ultrasonic probe is obliquely downward for vertical scanning, and the horizontal scanning is performed perpendicular to the position [2]. The ultrasonic probe is placed outward and upward to display. The supraspinatus tendon, where the patient's affected limb rests on the contralateral shoulder, is parallel to the supraspinatus of the scapula or longitudinal section, which can reveal the supraspinatus tendon and the teres minor tendon.

### 2.3. Diagnostic Criteria

A rotator cuff rupture includes a full-thickness rupture and a partial rupture. Ultrasound imaging of a full-thickness rupture shows loss of tendon or partial defect, and ultrasound imaging of a partial rupture shows local thinning or abnormal echo in the tendon.

### 2.4. Evaluation Indicators

All cases underwent shoulder joint mirror examination before ultrasound exploration, which is more sensitive and specific than ultrasound diagnosis.

### 2.5. Statistical Methods

Suppose a random vector comprises two parts: the visible part *Z* and the hidden part *C*, satisfying the distribution*P*(*C*, *Z*; *θ*). The training data are composed of several instances of Z. Now, *Z*={*z*_1_, *z*_2_,…, *z*_*m*_} the parameter *θ* needs to be estimated. To simplify the discussion, suppose that *C* is a discrete random variable that satisfies the distribution[*T*_1_, *T*_2_,…, *T*_*n*_], that is, *p*(*C*=*i*)=*T*_*i*_. The maximum like a lithoid estimate of *θ* is *θ*^*∗*^=arg max*P*(*Z|θ*) or *θ*^*∗*^=arg max*P*(*Z|θ*), but since *P*(*Z|θ*) may be very complicated, it is difficult to optimize.

When the function *f*(*x*) is convex, Jensen's inequality guarantees *f*(∑*T*_*i*_*x*_*i*_) ≥ ∑*T*_*i*_*f*(*x*_*i*_); among them, *T*_*i*_ ≥ 0, ∑*T*_*i*_=1. We can easily prove that the function log(*x*) is convex, so ln(∑*T*_*i*_*x*_*i*_) ≥ ∑*T*_*i*_ln(*x*_*i*_). Hence, the following equation is obtained:

(1) $$\begin{array}{c} \sum T_{i}x_{i}\geq \prod T_{i}x_{i}. \end{array}$$

We abbreviate *p*=(*C*=*i*, *Z|θ*) as *p*=(*C*_*i*_=*i*, *Z|θ*). From this, we have the following equation:

(2) $$\begin{array}{c} p=\left(Z|\theta \right)\sum p\left(C_{i},Z|\theta \right)=\sum \frac{p\left(C_{i},Z|\theta \right)}{C_{i}}T_{i}\geq \prod {\left[\frac{p\left(C_{i},Z|\theta \right)}{T_{i}}\right]}^{T_{i}}. \end{array}$$

The last inequality of equation (2) applies to equation (1). Take the logarithm of both sides of equation (2) to get the following equation:

(3) $$\begin{array}{c} \ln\;p=\left(Z|\theta \right)\geq \;\ln\;{\prod \left[\frac{p\left(C_{i},Z|\theta \right)}{T_{i}}\right]}^{T_{i}}=\sum_{i}T_{i}\left(\ln\;p\left(C_{i},Z|\theta \right)-\ln\;T_{i}\right). \end{array}$$

Let us write*L*(*θ*)=ln  *p*(*Z|θ*), *F*(*T*, *θ*)=∑_*i*_*T*_*i*_(ln  *p*(*C*_*i*_, *Z|θ*) − ln  *T*_*i*_); among them, *T*=(*T*_1_, *T*_2_,…*T*_*N*_)^*T*^. Formula (3) shows *L*(*θ*) ≥ *F*(*T*, *θ*)∀ *T*, *θ*. Besides, you can also understand the relationship between the *L* function and the *F* function in the following way:

(4) $$\begin{array}{c} F\left(T,\theta \right)=\sum_{i}T_{i}\left(\ln\;p\left(C_{i},Z|\theta \right)-\ln\;T_{i}\right)=\sum_{i}\left(T_{i}\ln\;p\left(C_{i},Z|\theta \right)-T_{i}\ln\;T_{i}\right)\\ =\sum_{c}p\left(c\right)\ln\;p\left(c,Z|\theta \right)-p\left(c\right)\ln\;p\left(C\right)T_{i}=p\left(C=i\right),p\left(c\right)=p\left(C=c\right)\\ =\sum_{c}p\left(c\right)\ln\frac{p\left(c,Z|\theta \right)}{p\left(c\right)}=-\sum_{c}p\left(c\right)\ln\frac{p\left(c\right)}{p\left(c,Z|\theta \right)}\\ =-\sum_{c}p\left(c\right)\ln\frac{p\left(c\right)}{P\left(c,Z|\theta \right)P\left(Z|\theta \right)}\\ =-\sum_{c}p\left(c\right)\ln\frac{p\left(c\right)}{p\left(c,Z|\theta \right)}+\ln\;p\left(Z|\theta \right)\\ =-\sum_{c}p\left(c\right)\ln\frac{p\left(c\right)}{p\left(c,Z|\theta \right)}+L\left(\theta \right)=-D\left(c\left\|c\right.|Z,\theta \right)+L\left(\theta \right). \end{array}$$

Among them, *D*(*p*‖*q*) is 2 Kullback–Leibler divergence distributed between *p* and *q*.

## 3. Results

### 3.1. Imaging Performance

The ultrasonic probe showed 10 cases of strange echo of the tendon and 5 cases of partial thinning. We explored 85 cases of local rupture and complete rupture of the supraspinatus tendon, and 25 cases of local rupture and complete rupture of the subscapular tendon. Our joint mirror examination results showed that 85 cases of local rupture and complete rupture of the supraspinatus tendon were detected [3]. 24 cases of local rupture and complete rupture of the subscapular tendon were also detected.

### 3.2. Diagnosis Results

All 50 patients were diagnosed with rotator cuff injury through pathological diagnosis, and their imaging examination results were compared with those of arthroscopy (Table 1).

**Figure 1:**
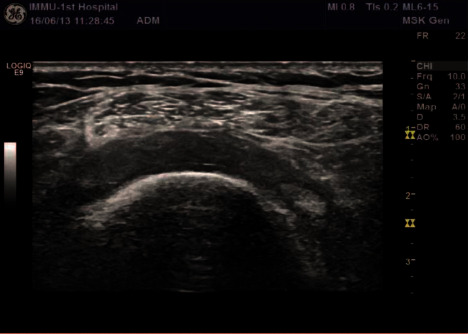
Two-dimensional ultrasound normal supraspinatus tendon.

**Figure 2:**
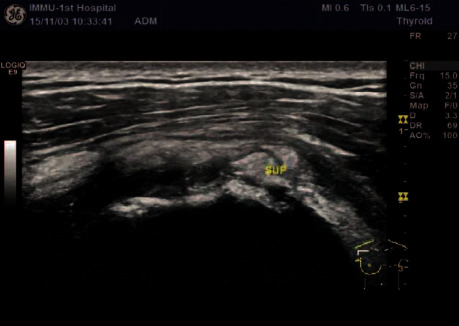
Two-dimensional ultrasound supraspinatus tendon injury.

**Figure 3:**
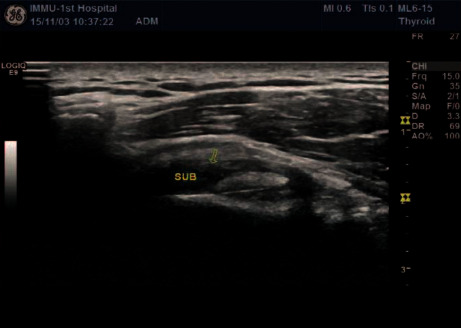
Two-dimensional ultrasound subscapular tendon injury.

**Figure 4:**
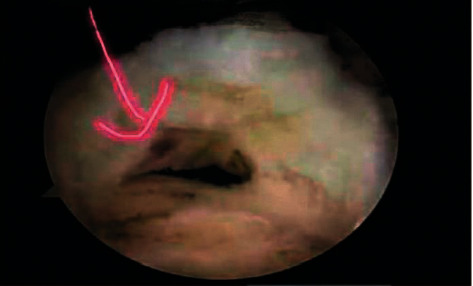
Intraoperative supraspinatus tendon arthroscopy.

**Figure 5:**
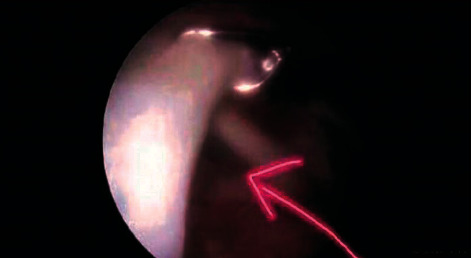
Intraoperative subscapular tendon arthroscopy.

A comparative analysis of the specificity and sensitivity of ultrasound examination and arthroscopy was conducted. Ultrasonography diagnoses five shoulders with a standard rotator cuff (Figure 1) and 95 shoulders with rotator cuff injury (Figures 2 and 3); 95 shoulders with rotator cuff injury diagnosed by arthroscopic surgery (Figures 4 and 5) and five shoulders with the standard rotator cuff. The overall sensitivity is 97.89%; the specificity is 60.00%; the positive predictive value is 97.89%; the negative predictive value is 60.00% (Table 2).

## 4. Discussion

There are many causes of shoulder pain. The most common causes are trauma, strenuous activity, excessive physical exertion, inflammation, and degeneration. These conditions can cause damage to soft tissues such as shoulder joint muscles and tendons. The rotator cuff is wrapped around the humerus and is a cuff-like tissue composed of the supraspinatus, supraspinatus, teres minor attached to the greater tuberosity of the humerus, and the subscapularis attached to the minor tuberosity of the humerus. In the function of human limbs, the rotator cuff mainly plays the role of supporting and stabilizing the scapulohumeral joint during joint shoulder movement and then maintaining the humeral head's normal fulcrum glenoid [4]. In short, in the joint shoulder movement, the rotator cuff plays a role in the decompression and stabilization of the humeral head. By analyzing the rotator cuff's anatomical characteristics, the study fully demonstrates that the rotator cuff's unique anatomical characteristics are the internal factors that cause the rotator cuff to be prone to injury and degenerative changes. Besides, the human body's external factors make the shoulder sleeve injury a frequently occurring joint disease in orthopedics. Many of the abovementioned anatomical characteristics are the internal factors that cause the rotator cuff tissue to be prone to damage and degeneration. Rotator cuff injuries are a frequently occurring disease.

In clinical practice, shoulder pain is a more common disease. Various clinical factors can cause shoulder pain, and rotator cuff injury is the main factor. In recent years, with the continuous development of the social economy and the continuous improvement of people's quality of life, sports are becoming more popular. With the promotion of the concept of sports for all, coupled with the arrival of the aging population, the requirements for shoulder joint function have become more and more critical. The higher the coming, the existence of such social factors also makes rotator cuff injury a common clinical disease. According to the statistical results of relevant clinical research data, the rotator cuff injury accounts for about 16.9% to 40.3% of the shoulder joint's various related diseases. Attention to rotator cuff injuries and early treatment is of great significance in preventing rotator cuff joint disease [5]. Through timely and correct diagnosis and early treatment, other joint and severe diseases caused by reduced shoulder joint movement function can be reduced. The primary incidence of rotator cuff injury is more common in young adults. Most of the injury causes are related to the supraspinatus muscle and its full-thickness injury during excessive lateral extension of the acromion. Timely and correct diagnosis and treatment of rotator cuff injuries can restore and prevent shoulder joint function. It is of great significance to reduce sickness and disability.

### 4.1. Imaging Methods for Diagnosis of Rotator Cuff Injury

Standard imaging methods for diagnosing rotator cuff injury include X-ray arthrography, MR, and ultrasound. When using an X-ray of the shoulder to examine the rotator cuff injury, if the contrast agent injected into the glenohumeral joint cavity overflows into the subacromial capsule or between the acromion and the supraspinatus muscle, it can be diagnosed as a complete rotator cuff injury. The specificity of the diagnosis of the rotator cuff's full-thickness injury is 100%, and the sensitivity is 98%. However, this method cannot detect nonfull-thickness rotator cuff injuries (40%–60% of symptomatic rotator cuff injuries), and there is a risk of contrast agent allergy and joint infection, especially in young male athletes, who are prone to vascular and vagal reactions. Besides, 48% of patients had different degrees of arthralgia 48 hours after the radiography.

The specificity and sensitivity of MR examination in diagnosing rotator cuff injuries are greater than 90%. However, abnormal signals such as damage to the hyperplastic granulation tissue around the rotator cuff, synovial fragments, or tendinitis can directly affect MR diagnosis accuracy. MR contrast examination (MRA) can increase the sensitivity and specificity to more than 93% and measure the area of damage. Still, this method is not used as a routine examination method, even in developed countries, and is only suitable for preoperative patients with particular diagnosis difficulties. The emergence of the 5 MHz linear array probe makes it possible to inspect rotator cuff injuries by ultrasound. At present, the specificity and sensitivity of ultrasound examination of rotator cuff injury are greater than 90%, which is similar to MR and MRA examinations.

The reason why ultrasound examination is suitable for diagnosing rotator cuff injury is that the water content of hyperplastic granulation tissue and synovial fragments is close to the broken end of the peripheral inflammatory reaction tendon plate. Still, the tissue density and reflection ability are quite different from it. Rotator cuff injury mainly involves the supraspinatus muscle, but the subscapularis or infraspinatus muscles can be affected when the injury is extensive. At this time, the RI injury is located at the junction of the sagittal plane and the coronal plane, which is difficult to detect by MR tomography. The ultrasound probe position can be tilted at any angle, making it possible to diagnose RI damage by ultrasound. Not only that, but the use of ultrasound diagnosis can also find other rotator cuff injuries other than the supraspinatus muscle and can also diagnose biceps prolonged head tendon diseases, so it has a unique value for follow-up after rotator cuff injury. Conventional ultrasound exploration is the most commonly used inspection method to find rotator cuff injuries. There are increasing reports of using ultrasound to explore the shoulder joint. Ultrasound exploration is adopted for shoulder injuries [6]. The sensitivity of the diagnosis of complete shoulder injuries is low, and the sensitivity to partial injuries diagnosis is relatively low. In this study, when ultrasound exploration was applied to rotator cuff injuries, not only local injuries of the supraspinatus tendon and subscapular tendon injuries could be diagnosed, but the imaging manifestations showed abnormal local echoes, mainly manifested as mixed echoes. During the patient's ultrasound examination, due to shoulder pain, the patient's tolerance was low, and he could not fully cooperate with the examination. Another reason was that the ultrasound examination operator failed to examine according to the patient's damage scope during the operation, and the history was insufficient. Different pathological changes in the rotator cuff tendon produce inflammation, fibrosis, granulation tissue hyperplasia, etc. These reasons may cause similar sonograms to appear. In this discussion, it was agreed that when 50 patients were diagnosed with ultrasound exploration, the sensitivity of ultrasound in supraspinatus tendon rupture was 100%, and the specificity was 55.55%. The diagnostic sensitivity of subscapular tendon rupture was 100%, and the specificity was 42.8%. The research evaluates that the use of ultrasound to detect rotator cuff injuries has obvious clinical value and should be used as the first choice to provide evidence for clinical treatment.

### 4.2. Factors Affecting Ultrasound Diagnosis of Rotator Cuff Injury

#### 4.2.1. Pain Factors

At present, the intensity of ultrasonic diagnostic equipment is below 13 MHz, which cannot penetrate bone tissue. Therefore, when inspecting the anterior rotator cuff injury, it is necessary to extend the shoulder joint so that the upper part of the supraspinatus muscle is exposed beyond the front edge of the acromion; when inspecting the posterior rotator cuff injury, it is necessary to flex the shoulder joint to make the supraspinatus muscle posterior. The upper part is exposed beyond the back edge of the acromion [7]. If the patient's shoulder pain is such that they are unable to perform flexion and extension exercises, or if the range of motion is reduced and they cannot maintain the flexion and extension position, it will affect the rotator cuff examination.

#### 4.2.2. Degeneration Factors

When the rotator cuff degenerates, different degrees of calcification can appear inside. At this time, the hypoechoic rotator cuff injury can be overlapped or masked by abnormal calcification.

#### 4.2.3. Other Factors

The accuracy of the ultrasound examination is also related to whether the operator has operational experience, knowledge of anatomy and pathology, immediate understanding and judgment ability, and the machine's sensitivity. Although the examinations in this group were performed by the same experienced ultrasound doctor and the same orthopedic clinician, there were still false positives in 2 patients in this group, which is considered related to this factor [8].

### 4.3. Ultrasound Imaging under Pathological Conditions of the Rotator Cuff

#### 4.3.1. Abnormal Rotator Cuff

**Figure 6:**
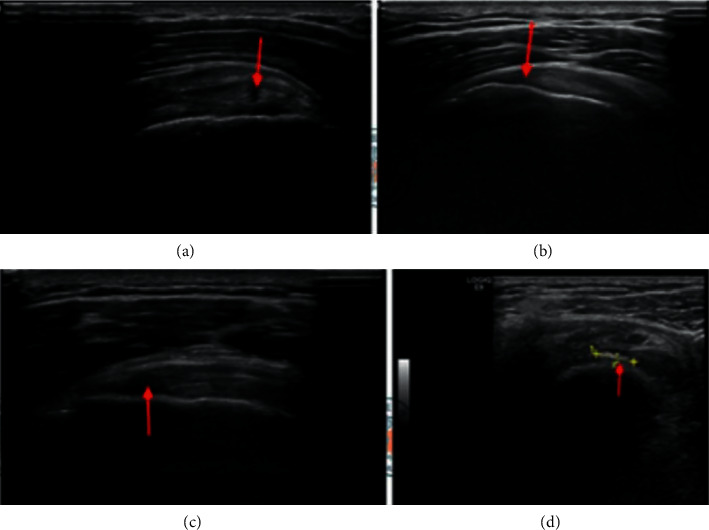
Image of the abnormal rotator cuff tendon. (a) Partial tear in the supraspinatus tendon. (b) Full -thickness tear of supraspinatus tendon. (c) Full -thickness tear of supraspinatus tendon. (d) Supraspinatus tendon cross-sectional calcification foci.

The main manifestations of rotator cuff abnormalities under ultrasound are partial tear, full-thickness tear, tendinitis, calcification, and impingement. Rotator cuff tears are most common in the supraspinatus tendon. Tendon tears can be divided into two types: partial tears and full-thickness tears. Partial tears generally occur locally in the tendon, mainly affecting the front third of the supraspinatus muscle. The longitudinal and cross-sectional ultrasound shows local small hypoechoic signals, as shown in Figure 6(a). If the tear has an extensive range, the irregular curvature of the tendon may be observed, and if there is a collapse, the size of the tear can be measured at the same time. Partial tears may occur on the bursal side, articular surface side, or inside the tendon. A full-thickness tear is directly penetrated from the side of the tendon and bursa to the side of the articular surface and is divided into small tears (3 cm). In an acute tear, the tendon retracts, but it has not entirely disappeared. Ultrasonic examination is more likely to find the tendon stump, as shown in Figure 6(b). In chronic tears, the end of the tendon disappears under the acromion. Under normal circumstances, a large area of the humeral head is not covered by the supraspinatus tendon, which is called the “naked-head” sign. Abnormal effusion in the acromion-deltoid subcapsular, glenohumeral joint, and acromioclavicular joint can be used as the second sign to judge a full-thickness tendon tear. After checking the supraspinatus tendon's full-thickness tear, further check the infraspinatus and subscapularis muscles, looking for large tears in each tendon. It is straightforward to distinguish the infraspinatus muscle from the humeral head's stop point [9]. It is only necessary to observe the dynamic observation during internal rotation and external rotation of the upper limb, as shown in Figure 6(c). Rotator cuff calcifications are common during ultrasound examination of the shoulder joint, and they are usually located at the stop point of the supraspinatus tendon. Calcified tendinitis can be divided into three stages, namely, precalcification, calcification, and late calcification. In the calcification phase, the calcification foci begin to reabsorb after a quiescence period after the formation of calcification, until the absorption enters the postcalcification stage. The echo signals of calcified sediments can be expressed as highly echogenic areas with clear boundaries, as shown in Figure 6(d). The fuzzy hyperechoic area (type II, the beginning of calcification reabsorption) and the lack of shadow (type III, the complete absorption of calcification) are severe calcification, which corresponds to the formation resorption process of calcification foci. Infraspinatus atrophy is another sign of tearing. Supraspinatus tendon tear is related to supraspinatus tendon tear, but subscapular tendon tear can occur alone. When the subscapular tendon is completely torn, the tendon's disappearance and the exposure of the humeral head can be seen under ultrasound. It is also possible to dynamically observe the tear of the subscapularis muscle when the upper limb is rotated internally and externally. A tear of the subscapularis is often accompanied by a dislocation of the biceps' long head tendon.

Ultrasound examination also has a high diagnostic value for tendinopathy and myopathy caused by chronic rotator cuff injuries and nerve injuries. The tendon injury caused by chronic rotator cuff injury manifests as the tendon's thinning, and the different degrees of calcification in the tendon are manifested as local echo enhancement [10]. Muscle atrophy caused by chronic tears or lack of innervation is manifested in ultrasound images as muscle thinning and echo reduction; muscle tissue fat infiltration is manifested as ultrasound texture blurring, and the echo is diffusely enhanced or decreased.

#### 4.3.2. Shoulder Impingement Sign

The shoulder joint's main impact types are divided into superior anterior impact or subacromial impact (the most common), anterior mid-direction impact, and superior posterior impact. The subacromial impact can be observed by dynamic exploration, and the probe should be placed on the lateral edge of the acromion in an oblique coronal position. During exploration, the upper limbs are first retracted, internally rotated, and then abducted. Under normal circumstances, the supraspinatus tendon and the acromion-deltoid subacromial sac move smoothly under the acromion without restricting movement and causing pain. During the examination, the shoulder is first in a neutral position, and then the glenohumeral joint is flexed to the maximum so that the supraspinatus tendon and the biceps long head tendon can collide with the anterolateral edge of the acromion. Secondly, the upper limbs were flexed 90°, adducted and internally rotated, and squeezed the supraspinatus tendon stop point and the subacromial capsule below the coracoacromial ligament.

#### 4.3.3. Abnormal Biceps Long Head Tendon

The most common abnormalities of the biceps long head tendon are tenosynovitis, tendon degeneration, tendon dislocation, and tear. Ultrasonic signs of tendon sheath inflammation of the long head of the biceps brachii muscle are as follows: swelling of the tendon sheath caused by hypoechoic or thickened tissue with missing echo signals, with or without local exudation and possible local Doppler vascular signs, and the abovementioned effects can be observed on two different cross-sections. Acute tenosynovitis is the widening of the tendon sheath without echo signal and the normal tendon's echo signal, while the typical chronic tenosynovitis is the uneven local signal of the tendon and the lack of signal of fiber texture. Hypoechoic and tendon thickening signals represent the ultrasound signal of long head tendonitis of the biceps brachii. When the long head of the biceps brachii tendon is dislocated, internodal grooves are empty and most tendons have shifted medially. The tendon's partial tear can be observed through the longitudinal section and the transverse section, that is, the hypoechoic signal area in the tendon. It is not difficult to make a clinical diagnosis of a complete rupture of the long head tendon: the retracted muscle can touch the soft tissue mass in the middle 1/3 of the upper arm, which is called the “Popeye” sign. Under normal circumstances, when examining the newly ruptured long head tendon of the biceps brachii, ultrasound should be able to find the two stumps of the ruptured tendon, which are often floating in the hematoma.

#### 4.3.4. Accuracy of Ultrasound Diagnosis

The wide application of ultrasonography in clinical practice has led to a lot of research on its diagnosis reliability. Studies have shown that surgeons' use of ultrasound training can improve their understanding of full-thickness rotator cuff tears. The value of ultrasound diagnosis depends on experienced sonographers, and to obtain reliable experience in diagnosing rotator cuff tears, sonographers must complete at least 100 ultrasound examinations of rotator cuff tears [11]. After training, the ultrasound diagnosis of full-thickness rotator cuff tears differs little among technicians, but the diagnosis of partial tears and internal tendon tears differs significantly.

Studies have shown that when evaluating a full-thickness rotator cuff tear, ultrasound examination is equivalent to MRA. Still, when evaluating a partial rotator cuff tear, ultrasound examination is inadequate, especially for tears less than 1 cm, and the sensitivity and specificity of ultrasound are significantly reduced. A meta-analysis pointed out that professional ultrasound training can improve the diagnostic level of rotator cuff tears, especially the sensitivity and specificity of the diagnosis of full-thickness tears can reach 92.3% and 94.4%, respectively, while partial tears can only reach 66.7% and 93.5%, respectively. Ultrasonography can make a correct judgment on the standard or completely ruptured long head tendon of the biceps brachii, but it is not accurate in assessing partial tears of the long head tendon of the biceps brachii tendon due to degeneration and tendinitis. Another study found that 75% of cases diagnosed as tendinitis by ultrasound were confirmed as partial tears during the operation. In assessing the size of a rotator cuff tear, ultrasound underestimated the size of the rotator cuff tear on average by about 15 mm, while the underestimation of MRA was about 4 mm on average. Therefore, ultrasound can be used to investigate patients suspected of having a rotator cuff tear. If the ultrasound is positive, MRA can be considered. At the same time, ultrasound is used to evaluate and grade the rotator cuff's fat infiltration. Its value is equivalent to that of MRI. It can be used as a preliminary examination method for fat degeneration of the rotator cuff muscles.

#### 4.3.5. Ultrasound-Guided Puncture Diagnosis and Treatment

Ultrasound-guided puncture-assisted diagnosis and treatment have received increasing attention. A group of controlled clinical studies on 145 patients showed that ultrasound-guided puncture treatment of chronic subacromial bursitis could improve the efficacy, especially the passive range of movement of the affected limb. However, a meta-analysis pointed out that the difference between ultrasound-guided puncture and blind puncture is statistically significant in improving patients' pain and increasing shoulder movement, but it has no clinical significance. Calcified tendinitis is the cause of a considerable number of patients with shoulder pain, and ultrasound-guided lavage and extraction of calcified deposits to treat calcified tendinitis have the advantages of fewer complications and significant clinical effects. It has become a more effective solution to this cause of shoulder pain treatment plan.

## 5. Conclusion

In summary, ultrasound is efficient in detecting abnormal changes in the shoulder joint and its surrounding soft tissues. It adopts ultrasound exploration for patients with rotator cuff injuries. It has high diagnostic sensitivity and is a reliable and effective clinical diagnosis method. It is worthy of clinical diagnosis. Application and promotion is carried out in China.

## Figures and Tables

**Table 1:** Comparative analysis of the specificity and sensitivity of the two diagnostic methods for tendon injury.

Result	Ultrasound inspection method		Arthroscopy	
	Supraspinatus tendon tear	Subscapular tendon tear	Supraspinatus tendon tear	Subscapular tendon tear
Specificity	85/85 (100.00)	30/30 (100.00)	85/85 (100.00)	30/30 (100.00)
Sensitivity	25/45 (55.55)	12/28 (42.80)	40/45 (90.00)	24/28 (85.70)

**Table 2:** Comparison of the inspection results of the two diagnostic methods.

	Positive	Negative	Total
Ultrasound diagnosis	95	5	100
Arthroscopy	95	5	100

## Data Availability

The data used to support the findings of this study are available from the corresponding author upon request.
